# Chemistry of Silanes: Interfaces in Dental Polymers and Composites^1^

**DOI:** 10.6028/jres.110.081

**Published:** 2005-10-01

**Authors:** Joseph M. Antonucci, Sabine H. Dickens, Bruce O. Fowler, Hockin H. K. Xu, Walter G. McDonough

**Affiliations:** National Institute of Standards and Technology, 100 Bureau Drive, Stop 8545, Gaithersburg, MD 20899 USA; American Dental Association Foundation, Paffenbarger Research Center, 100 Bureau Drive, Stop 8546, Gaithersburg, MD 20899 USA; National Institute of Standards and Technology, 100 Bureau Drive, Stop 8545, Gaithersburg, MD 20899 USA; American Dental Association Foundation, Paffenbarger Research Center, 100 Bureau Drive, Stop 8546, Gaithersburg, MD 20899 USA; National Institute of Standards and Technology, 100 Bureau Drive, Stop 8545, Gaithersburg, MD 20899 USA

**Keywords:** adhesion, interfacial adhesion, interphases, silanes, silanization, silsesquioxanes

## Abstract

The performance and service life of glass-or ceramic-filled polymeric composites depend on the nature of their resin, filler and interfacial phases as well as the efficacy of the polymerization process. The synergy that exists between the organic polymer matrix and the usually inorganic reinforcing filler phase is principally mediated by the interfacial/interphasial phase. This latter phase develops as a result of the dual reactivity of a silane coupling agent, (YRSiX_3_), a bifunctional molecule capable of reacting with the silanol groups of glass or ceramic fillers via its silane functional group (–SiX_3_) to form Si-O-Si- bonds to filler surfaces, and also with the resin phase by graft copolymerization via its Y functional group, usually a methacrylic vinyl group. In this paper, we explore some of the chemistry of organosilanes, especially that of functional organosilanes (or silane coupling agents as they are commonly known) that are used to mediate interfacial bonding in mineral reinforced polymeric composites. The chemistry of organosilanes can be quite complex involving hydrolytically initiated self-condensation reactions in solvents (including monomers) that can culminate in polymeric silsesquioxane structures, exchange reactions with hydroxylated or carboxylated monomers to form silyl ethers and esters, as well as the formation of silane derived interfaces by adhesive coupling with siliceous mineral surfaces.

## 1. Introduction

Except for pure gold fillings, all dental restoratives are multiphase materials having a composite microstructure involving one or more interfaces or interphases. With regard to composites, the term interface is reserved for the relatively sharp boundary layer that exists between the continuous or matrix phase and the dispersed or filler phase of these heterogeneous materials. In many composites, however, the microstructure is characterized by a broad, more gradient-like transition zone that forms between the continuous and dispersed phases that is more accurately referred to as an interphase [1–9]. For example, this diffuse type of inter-phase is characteristic of acid-base type dental cements, e.g., carboxylate and glass-ionomer cements, especially the latter. The sharp type interface is more characteristic of amalgams and resin-based, macro-sized glass or ceramic filled composites.

The modern development of dental composites owes much to R. L. Bowen of the American Dental Association for his pioneering studies at the National Bureau of Standards, currently the National Institute of Standards and Technology. His recognition of the excellent matrix forming potential of epoxy resins as well as their poor ambient polymerization characteristics (slow under anionic catalysis and uncontrollable under the more rapid cationic catalysis then available) led him to the discovery of a unique hybrid monomer which combined the low polymerization contraction of epoxy resins with the excellent setting behavior of acrylic monomers [10]. His classical synthesis of the bulky, thermosetting dimethacrylate, Bis-GMA, 2,2-bis[p(2′-hydroxy-3′-methacryloxypropoxyphenyl)]-propane, his preparation of silica fillers that combined translucency and radiopacity while matching the refractive indices of the resin matrix, and his utilization of the technology of silane coupling agents, ushered in the modern era of esthetic dental composites [11,12].

## 2. Overview

### 2.1 Polymeric Dental Composites

Polymeric dental composites are interconnected heterogeneous materials that generally have three discernable phases: (1) a polymeric matrix or continuous phase formed by polymerization of a resin system consisting of one or more monomer/oligomers activated for chemical/photochemical polymerization, (2) a higher modulus dispersed phase consisting of fillers of various types (silica, ceramic, organic, etc.), sizes, shapes and morphologies, and (3) an interfacial or interphasial phase that bonds to both the continuous and dispersed phases, thereby enhancing the moduli and mechanical properties of the weaker polymer phase and also facilitating stress transfer between these phases by forming a unitary material. Polymerization adhesion of lower moduli polymeric matrices to higher moduli inorganic fillers can occur as a result of van der Waals forces, ionic interactions, hydrogen bonding, ionic or covalent bonding, interpenetrating polymer network formation and, for certain types of fillers, by micromechanical interlocking mechanisms. For most mineral reinforced dental composites, the primary interphasial linkage between the polymer matrix and the filler phase is by chemical bond formation, mediated by a dual functional organosilane, termed a silane coupling agent.

### 2.2 Chemistry of Organosilanes

**Fig. 1:**
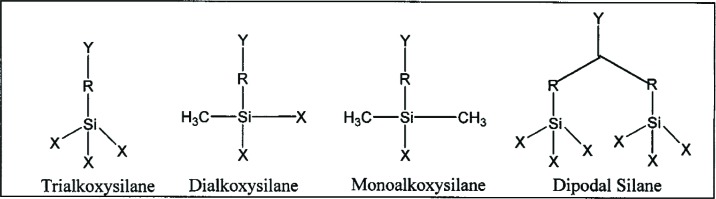
Generic structures of functional organosilanes with varying numbers of hydrolyzable substituents on silicon.

**Fig. 2:**
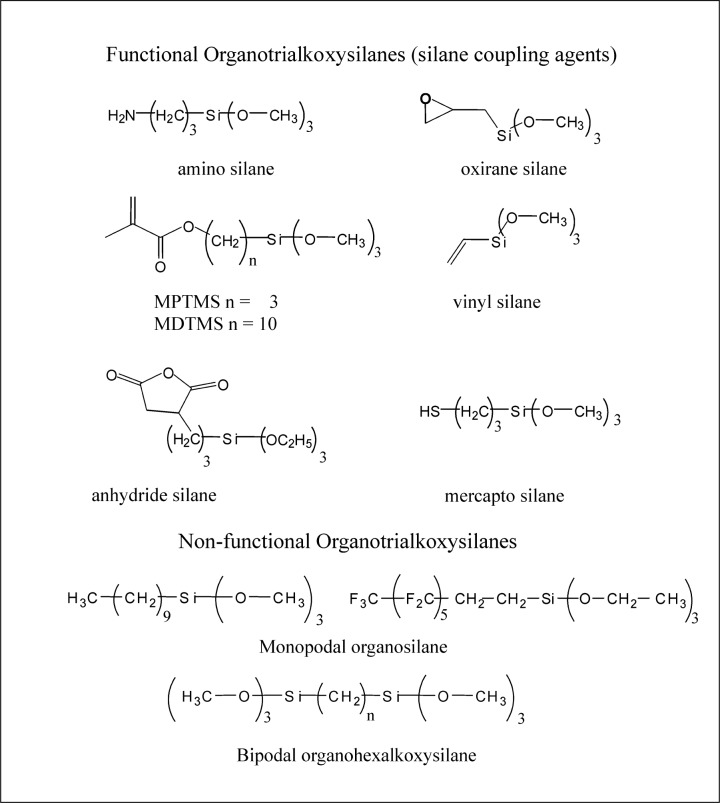
Functional and non-functional organoalkoxysilanes.

Organosilanes, (R_1_R_2_R_3_)SiX*_n_* (where *n* = 1 to 3), are a unique class of organic silicon compounds that have a hydrolytically active silicon based functional group, SiX*_n_*. They can react with both inorganic and organic substrates as well as with themselves and other silanes by complex hydrolysis-condensation reactions to form a variety of hybrid organic-inorganic structures. The R groups in organosilanes can be non-reactive substituents, e.g., hydrocarbon or fluorocarbon chains; these types of silanes are frequently used as release agents for the protective coating of various substrates. Also, the R groups can be reactive substituents with terminal functional groups capable of specific chemical reactions, e.g., a methacrylate or epoxy (oxirane) group that will copolymerize with methacrylic or epoxy monomers, respectively. These latter types of organosilanes have dual functionalities and are referred to as silane coupling agents. They can be represented by the general formula, Y-(R_1_R_2_R_3_)-SiX*_n_*, where R_1_, R_2_ and R_3_ are organic substituents that can be the same or different, but at least one has a reactive functional group, *Y*, as well as a SiX*_n_* group capable of bonding to siliceous surfaces, (*n* = 1 to 3). The X substituents on silicon, e.g. –Cl, –NH_2_, –OCH_3_, –OCH_2_CH_3_, or esters such as –O_2_CCH_3_, are usually readily hydrolyzable, so that the trifunctional –SiX_3_ group of RSiX_3_ can form RSiX_2_OH, RSiX(OH)_2_, or RSi(OH)_3_ silanol intermediates by reaction with water. Most silane coupling agents are usually trifunctional (Y-R-SiX_3_) with respect to their hydrolyzable substituents; X is usually an alkoxy substituent, e.g., –OCH_3_, –OCH_2_CH_3_. However, the silicon functional group also can have two or just one X substituent, i.e., Y-R_1_R_2_SiX_2_ and YR_1_R_2_R_3_SiX. The monoalkoxysilane can only form a monolayer, whereas the trialkoxy- and dialkoxysilanes (after hydrolysis to their silanol forms) lead to multi-layered interphases. Generic structures of these types of organosilanes are illustrated in Figs. 1 and 2, along with a dipodal silane with two trifunctional silane groups per molecule [7–9].

### 2.3 Interactions of Silanes With Siliceous Fillers

**Fig. 3:**
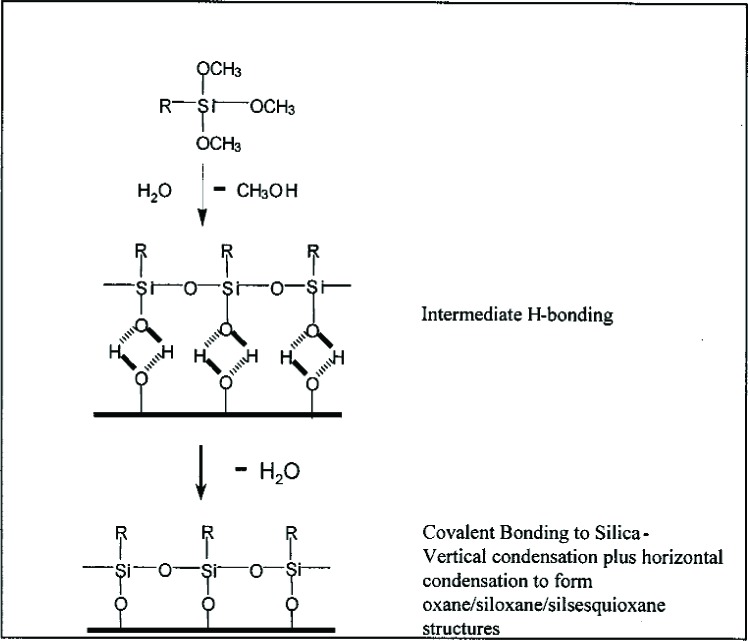
Simplified reaction of organotrialkoxysilanes with silica surfaces.

Fig. 2 shows specific chemical structures of functional and non-functional organoalkoxysilanes (silane coupling agents). Figure 3 displays an idealized reaction of a trialkoxysilane with a substrate having silanol groups showing vertical condensation to form covalent bonds to the substrate as well as horizontal condensation to form polymeric siloxane structures. These organosilane intermediates, in the absence of substrates such as silica or similar minerals, or in the absence of hydroxyl-, amino- or carboxylic acid-containing organic compounds, undergo a complex series of hydrolysis and self condensation reactions leading to dimers, trimers, tetramers and ultimately oligomers and polymers designated as silsesquioxanes, [RSiO_3/2_]*_n_*. Conditions (catalyst, solvent, temperature) of the silanization process used to affect the complex hydrolysis-condensation reactions of organosilanes will determine the nature of the products formed. Therefore, for a given organosilane and substrate, the silanization method also will determine what type of silane-derived species forms on the surface of composite fillers. As expected, the interfacial layer on silanized fillers is likely to be more complicated than that depicted in Fig. 3 and usually is multilayered, comprising both chemically and physically adsorbed species [2–9, 13–21].

### 2.4 Silane-Derived Interfaces/Interphases in Dental Composites

In methacrylic resin based dental composites, adhesion between the polymeric matrix and the reinforcing filler is usually achieved by use of the silane coupling agent, 3-methacryloxypropyltrimethoxysilane (MPTMS), a bifunctional molecule capable of reacting via its alkoxysilane groups with the filler and itself, and with the resin by virtue of its methacrylate functional group. The overall degrees of reaction of the silane with the glass filler (oxane bond formation), with itself (by siloxane formation), and with the resin system (by graft copolymerization) determine the efficacy of the coupling agent. The oxane bond (silicon-oxygen-silicon) that forms between the silane agent and the mineral filler can be especially vulnerable to hydrolysis, because this covalent bond has significant ionic character [1–9, 13–28]. By contrast, the carbon-carbon covalent bond that forms between the silane and the polymer matrix is considerably more stable to hydrolytic attack than the silicon-oxygen covalent bond. For a given resin/filler system, the physical-chemical nature of the silane agent, (e.g., chemical structure, molecular size, degree of hydrophobicity, reactivity, functionality), the silanization procedure employed, the silane layer orientation that develops and the extent of filler coverage are important parameters that determine many of the physicochemical and mechanical properties of the interphase, and in turn, those of the composite. The durability of the interface/interphase in the oral environment and its ability to transfer stresses between the polymer and filler phases during mastication are especially important properties for dental composites to have. One approach aimed at improving the quality and durability of the filler/matrix interface involves the use of more hydrophobic and flexible silane coupling agents than MPTMS [22–24].

### 2.5 Silanization Methods

In commercial dental composites, the particulate glass fillers are usually presilanized by deposition from dilute aqueous/organic solutions of MPTMS. Bulk deposition of the silane by spray-on techniques during mechanical mixing or grinding of the fillers is also employed. The treated glass fillers are then incorporated by mechanical mixing into a resin phase that has been activated for subsequent chemical or photochemical polymerization of the resulting composite paste. For optimal composite restorative properties such as high modulus and strength, the highest filler content consistent with a workable paste rheology is sought. How well the filler blends with the resin system, depends on the nature and composition of the resin component, the nature of the glass/ceramic filler (type, size, shape, surface area, etc.) and the type of silane coupling agent. The silanization procedure used to coat the filler phase is also an important factor in determining interfacial properties, e.g., extent of coverage of the silane-derived coating that is formed on the surface of the glass. When applied from solvents the type of solvent (polar vs non-polar) and the catalyst (acid vs base), and the type and amount of silane agent, amount of water, pH, temperature, etc., are all important factors in determining the type of interphase generated [8,9,13].

The well-known reaction of silanes with glass or ceramic substrates can be quite complex, depending on the chemical structure of the silane and the silanization process. Organosilanes can, by hydrolysis-condensation reactions, interact with surface silanols of the mineral (vertical condensation), react with themselves to form siloxanes by horizontal condensation and, in the case of trifunctional silanes of the type RSiX_3_, form silsesquioxanes by three-dimensional condensation. With R_3_SiX, which can only form single oxane bonds with the substrate or a siloxane dimer by reaction with itself, only a monolayer interface is possible. Similar to the behavior of RSiX_3_, silanes with the structure R_2_SiX_2_ also can condense both with surface silanols via oxane bonds and intermolecularly via siloxane bonds, thereby tending to form multi-layered interfaces.

For the silanization of siliceous fillers for use with the new epoxy dental resins, organosilanes having the epoxy or oxirane functionality rather than vinyl groups are employed (Fig. 2). Silanization of fillers with epoxy silane coupling agents requires avoidance of acidic or basic conditions to avoid hydrolytic opening of the oxirane functionality. Because of the vulnerability of the oxirane group to attack by H_2_O (especially in the case of bicyclic oxirane silane coupling agents such as 2-(3,4-epoxycyclohexyl)ethyltrimethoxysilane), it also would seem prudent to use hydrophobic epoxy resins in the formulation of oxirane-based composites [29].

An attractive alternative to the above presilanization methods for surface activation of the filler phase of composites is *in situ* silanization of glass fillers [8,17,30]. This technique, also referred to as integral blending, is simpler than presilanization and involves simply adding the silane agent, e.g., MPTMS, as a comonomer to the usual dental monomer systems, and then forming a composite paste by admixture with unsilanized glass filler. While it has been shown (indirectly by mechanical tests) that *in situ* silanization provides effective coupling of the glass and polymer matrix phases, it is not clear that adhesion is only the result of the silanol groups of the glass filler (Fig. 3) and the –Si(OCH_3_)_3_ or –Si(OH)_3_ groups of MPTMS reacting to form oxane linkages. By-products from *in situ* silanization such as water and alcohols conceivably can lead to voids in the composite structure. Because of the low temperature employed in this method and the presence of organic sites for reaction in some resins, this mode of silanization may adversely affect the rheology and other properties of the *in situ* silanized composite paste (see interaction of organosilanes with Bis-GMA and other dental monomers). The best evidence for silaneglass chemical bonding involves studies with presilanized glasses where it was shown, by both infrared and Raman spectroscopic studies, that chemical reactions occur between the surface silanols and silanol groups of the coupling agent. Presumably these reactions involve prior formation of hydrogen bonded intermediates that then eliminate H_2_O or CH_3_OH to form covalent Si-O-Si bonds to the glass surfaces (Fig. 3).

## 3. Recent and Current Organosilane Research at NIST: Methology, Results, and Discussion

### 3.1 Effects of Silane Coupling Agent and Filler Type on Composite Strength

In order to enhance our understanding of how the chemical structure of silane coupling agents can affect the properties of dental composites, we investigated the effects of increasing the hydrocarbon-connecting segment of the methacrylic silane coupling agent from three to ten methylene units as shown in Fig. 2 [26,27,31]. The increase in length of the connecting segment of the silane agent is expected to introduce enhanced hydrophobicity and flexibility into the interphase. In addition, it is likely that hydrophobic monomer systems would show greater compatibility with these more hydrophobic silanized fillers. Hydrophobic resin-based composites exhibit low water uptake and enhanced durability in aqueous environments [1–38], and the addition of filler surface treated with reactive, hydrophobic silanes is expected to augment these properties [27,28,31].

#### 3.1.1 3-methacryloxytrimethoxysilane (MPTMS) and 10-methacryloxydecyltrimethoxy-silane (MDTMS)

Four experimental photo-polymerizable composites were prepared from a resin system consisting of equal masses of 2,2-bis[p-(2′-hydroxy-3′-methacryloxy-propoxy)phenyl]propane (Bis-GMA) and triethylene glycol dimethacrylate, TEGDMA [27]. A barium boroaluminosilicate glass powder (particle size: 0.94 µm) and a crushed quartz (particle size: 28 µm) were selected as fillers, and silanized with either MPTMS or MDTMS (10-methacryloxydecyltrimethoxysilane; Fig. 2). Equivalent amounts of each silane were applied to the glass powder from cyclohexane with n-propylamine (2 % by mass fraction based on the mass of the filler) as the catalyst. Rotary evaporation was used to remove the solvent and by-products and the silanized fillers were heated at 80 °C (± 5 °C) for 30 min and then at 100 °C(± 5 °C) for 30 min. MDTMS is expected to be more hydrophobic than MPTMS because of its greater hydrocarbon content. The composite pastes were formulated with a mass fraction of 75 % of the silanized fillers. Twenty (2 × 2 × 25) mm rectangular bar-shaped specimens of each composite were prepared for the 3-point flexural test. The specimens were stored in distilled water at 37 °C for 24 h and then half were additionally stored in distilled water at 90 °C (± 5 °C) for another 408 h. The mean values and the standard deviations of the flexural strengths (MPa) of the four composites are summarized in Table 1 (number of specimens = 10).

The flexural strengths were analyzed using 3-way ANOVA at the 95 % confidence level. The main factors (filler types, coupling agent and storage condition) were significant. In addition, the interaction between coupling agent and storage condition was significant; however, the interaction between the filler type and storage condition was not. Similar results, shown in Table 2, were obtained using equivalent amounts of MPTMS vs MDTMS on a larger size (45 µm) barium boroaluminosilicate glass filler that was blended with photoactivated Bis-GMA/TEGDMA, 1:1 by mass [31]. These results showed the same trends of a previously discussed microbond test study comparing MPTMS and MDTMS and suggest that the durability of dental composites in aqueous environments can be improved by the use of hydrophobic coupling agents such as MDTMS [22–24]. A further advantage of using fillers silanized with MDTMS is that this surface treatment enables higher filler loadings to be achieved as shown for the last two MDTMS-based composites in Table 2 [31].

#### 3.1.2 Structural Variations in the Number of Y and/or SiX_3_ Functionalities

**Fig. 4:**
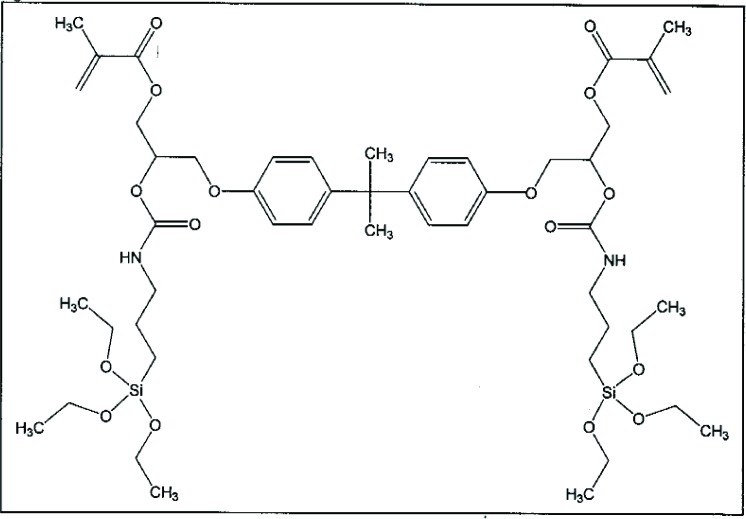
A multifunctional silane agent derived from Bis-GMA and 3-isocyanatopropyltriethoxysilane. This type of silane agent conferred enhanced hydrolytic durability on glass filled composites when compared with similar composites filled with the same glass treated only with MPTMS.

In addition to altering the chemical structure of silane coupling agents through varying the length and structure of the segment R in Y-R-SiX_3_, structural variations also can be made in the number of Y and/or SiX_3_ functionalities. Such a multi-functional organosilane is shown in Fig. 4. The multifunctional silane agent was derived from the reactions of Bis-GMA and 3-isocyanatopropyltriethoxysilane. This multi-functional bipodal type of silane agent conferred enhanced hydrolytic durability on glass-filled composites when compared with similar composites filled with glass treated with MPTMS only, presumably because of the increased degree of crosslinking in the interphase [17].

#### 3.1.3 Substrate Effects

The surface nature of the inorganic substrate or filler must be considered in the selection of a silane coupling agent [1–9,14–16,39–41]. Factors to be considered include the type and availability of surface hydroxyl groups (silanol vs adsorbed water, hydrated ions, etc.), hydrolytic stability of the oxane bond that forms, number of active hydroxyl groups per unit area of substrate, surface reactivity and chemical/physical properties of the silane and silanization conditions (amount of silane, method of deposition, pH, temperature, type of catalysis, etc.). With silica, quartz, E-glasses, boroaluminosilicates, and zirconia silicates sufficient surface silanol groups are present in these substrates to enable effective silanization to occur to yield well surface-modified fillers that then reinforce the polymer matrices of composites. Substrates that are not readily amenable to the usual silane coupling agents include calcium salts, e.g., oxides, carbonates, phosphates and alkali glasses such as sodium glasses [1–9,14–16]. Glasses with high alkali or phosphate contents not only do not form stable oxane bonds with silane coupling agents, but they also can catalyze the disruption and redistribution of Si-O-Si bonds in the silane-derived horizontally condensed products. To effect bonding to these types of substrates, modification of the substrate or the use of complex silane systems may be necessary. A recent example of improved silanization of silica nitride whiskers (a substrate that does not silanize well) by surface modification of the substrate is described below.

#### 3.1.4 Ceramic Whisker Reinforcement of Composites

A unique approach to substrate modification was recently taken with the use of ceramic whiskers as fillers for dental resins [40,41]. The ceramic whiskers are single crystals possessing a high degree of structural perfection and, as a result, very high strength values ≈ 30 GPa. For comparison, the strength of polished bulk glass is about 0.1 GPa, and that of glass fibers is ≈ 3 GPa. The fracture toughness of crystalline ceramics (silicon nitride, alumina, zirconia, etc.) ranges from about (2 to 6) MPa · m^1/2^, while that of glass is only about 0.8 MPa · m^1/2^. In addition, the shape of the whiskers is highly elongated (e.g., a diameter of 0.5 µm and a length of 5 µm) with the potential benefit of being more effective in bridging a microcrack and preventing it from propagating and in resisting dislodgement from the matrix during wear. While extensive studies exist on fiber reinforcement of dentures and retainers, the fibers differ from the whiskers in that the fibers are usually polycrystalline or amorphous, while the whiskers are single-crystalline. The strength of the whiskers is about 10 times that of the fibers, while the size of the whiskers is orders of magnitude smaller. Furthermore, the properties of a fiber composite are usually anisotropic and heterogeneous depending on fiber size and orientation suitable for structural applications. The whisker composites have relatively isotropic and homogeneous properties and are more suitable for contact and wear applications. In the past, ceramic single-crystalline whiskers have been used to reinforce ceramics and metals but not resin matrices for dental composites. Single-crystalline silicon nitride (*β*-Si_3_N4) whiskers with a diameter ranging from 0.1 µm to 1.5 µm with a mean of 0.4 µm, and length ranging from 2 µm to 20 µm with a mean of 5 µm were initially investigated as reinforcements for dental resins. Compared to silica type fillers, these whiskers (*β*-Si_3_N_4_) lack sufficient surface SiO_2_ with silanol groups to effectively interact with silane coupling agents during silanization. As a consequence, composites with these fillers, even when silanized, were inferior to similar composites formulated with silanized glass fillers [40,41].

**Fig. 5:**
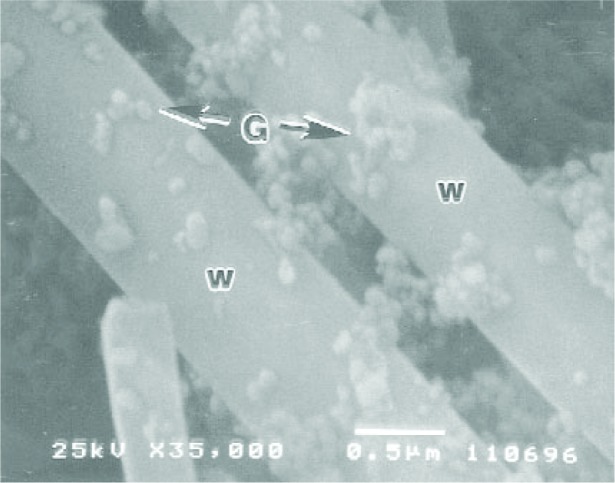
SEM micrograph of nano-sized silica glass particles “G” fused onto the silicon nitride whiskers “w” by heating at a temperature of 800 °C. The purpose of the silica-whisker fusion was to facilitate the silanization of whiskers and roughen the whisker surfaces for enhanced retention in the resin matrix.

A key challenge in whisker reinforcement is to improve the whisker-matrix bonding. Fusing submicrometer silica glass particles onto the individual whiskers has the benefits of (1) facilitation of silanization regardless of the whisker composition (e.g., silicon carbide, sapphire, zirconia, mullite); and (2) enhancing whisker retention in the matrix by providing rougher whisker surfaces (Fig. 5). Fusing silica particles onto silicon nitride whiskers at 800 °C significantly improved the composite mechanical properties over similar composites utilizing silanized untreated whiskers or whiskers silanized after the thermal oxidative treatments. Fusion of silica onto *β*-Si_3_N_4_ at 650 °C did not improve the composite properties, perhaps due to insufficient softening of the silica particles. The composite properties became inferior for silica*β*- Si_3_N_4_ fused at 1000 °C, probably a result of whisker degradation at this temperature. The mechanisms of glass fusion as a function of the type of glass and substrate are deserving of further exploration.

#### 3.1.5 Formation of Silsesquioxanes

**Scheme 1:**
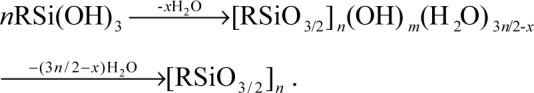
Pathway to incompletely- and fully-condensed silsesquioxanes

Organosilsesquioxanes, also known as T-resins, are unique hybrid organic-inorganic oligomeric or polymeric materials that have complex cyclic, cage-like structures [42–46]. The generic structure of the organosilsesquioxanes is given by the empirical formula (RSiO_1.5_)*_n_*, where the stoichiometric ratio of oxygen to silicon is 1.5 (sesqui), R is the organic substituent and *n* is the number of mer units in the oligomer. Early studies involving condensation reactions of silicic acid, Si(OH)_4_, assigned silsesquioxane structures to some of the oligomeric products of this inorganic acid. The actual structure designated by (RSiO_1.5_)*_n_* can be quite complex and may include many polycyclic forms, e.g., polyhedral, ladder, semi-ladder, highly branched and other amorphous forms. Because of their high Si-O-Si contents that results from the loss of alkoxy groups and a series of silanol/silanol or silanol/silyl ether condensation reactions, these resins bear a general resemblance to silica and potentially have properties suitable for use in polymeric dental materials, both as components of the resin matrix phase or as organically modified silica fillers with molecular dimensions. Scheme 1 illustrates the hydrolytically catalyzed condensation reactions of RSi(OCH_3_)_3_ to form incompletely-condensed silsesquioxanes, where *m* ≪ *n*, and fully-condensed silsesquioxanes, [RSiO_3/2_]*_n_* [42–44].

#### 3.1.6 Synthesis and Characterization of Oligomeric MPTMS (OMPTMS)

**Fig. 6:**
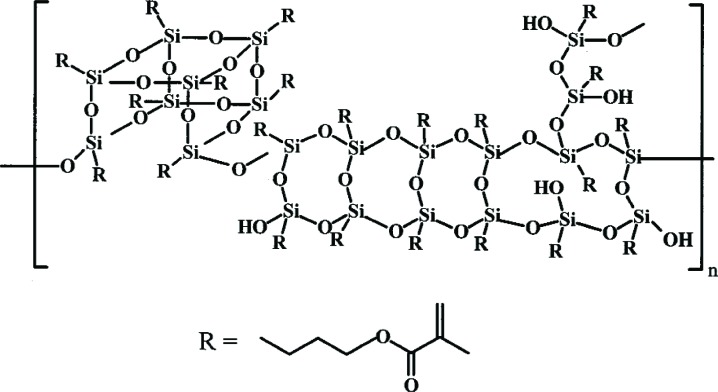
Generalized structure of three-dimensional silsesquioxanes formed from MPTMS and water as determined by MALDI-TOF mass spectrometry. The essential difference between silsesquioxane oligomers and polymers is the level of –SiOH condensation.

Several controlled hydrolysis-condensation reactions of MPTMS in catalyzed and non-catalyzed aqueous organic solvents were conducted at 23 °C to 70 °C. For example, a homogeneous solution of 42 % MPTMS, 11 % H_2_O, and 46 % acetone with 1 % *n*-propylamine as the catalyst (all percentages are on a mass fraction basis) was allowed to concentrate over a period of 3 d at 23 °C. After removal of residual solvent and by-products, a clear pale yellow, viscous liquid OMPTMS was isolated in > 90 % yield. Matrix-assisted laser desorption/ionization time-of-flight (MALDI-TOF) mass spectrometry was used to deduce the three dimensional structure of a complex silsesquioxane polymer [44]. A simplified structure of the silsesquioxanes formed by the reaction of MPTMS in aqueous media is shown in Fig. 6.

Similar reaction conditions to those described above were used to prepare the liquid oligomer (OMDTMS) derived from 10-methacryloxydecyltrimethoxysilane (MDTMS). Acid catalysis (acetic, formic, or hydrochloric acid) was also effective in the synthesis of polymeric silsesquioxanes from organosilanes. Liquid films of photoactivated OMPTMS and OMDTMS were polymerized with a visible light source to yield clear polymer films. The oligomers, OMPTMS and OMDTMS, were insoluble in water but soluble in many organic solvents and in all common acrylic monomers. Photoactivated OMPTMS and OMDTMS polymerized by exposure to visible light irradiation for one min, formed clear, hard glassy solids that appeared to be crosslinked because of their insolubility in common organic solvents. By contrast, the polymerization of MPTMS or MDTMS was sluggish and formed soft, readily soluble oligomers after 10 min irradiation with visible light. NMR and infrared spectroscopy are well suited to characterize the features of MPTMS and MDTMS, and their conversion to the liquid oligomers, OMPTMS and OMDTMS. The most notable features in the ^1^H NMR spectra of the silsesquioxanes (not shown) are related to the methoxysilane group. As the reaction proceeds, the sharp trimethoxy signal of the unreacted silane at 3.6 ppm (parts per million, a customary unit in NMR spectroscopy, see J. Mc Murry, Organic Chemistry, Brooks/Cole Publishing Co. (1988) p. 415) was replaced by a broader methoxy signal at 3.5 ppm (expanded uncertainty, 0.02 ppm) due to partially hydrolyzed and reacted silane. The methoxy signal gradually disappears and the remaining peaks in the spectra all show significant broadening that is indicative of a relatively high molecular weight product. Integration of the spectra verifies no hydrolytic loss of the methacrylic ester or premature polymerization of the pendant methacrylate functional groups.

##### 3.1.6.1 IR Analyses

**Fig. 7:**
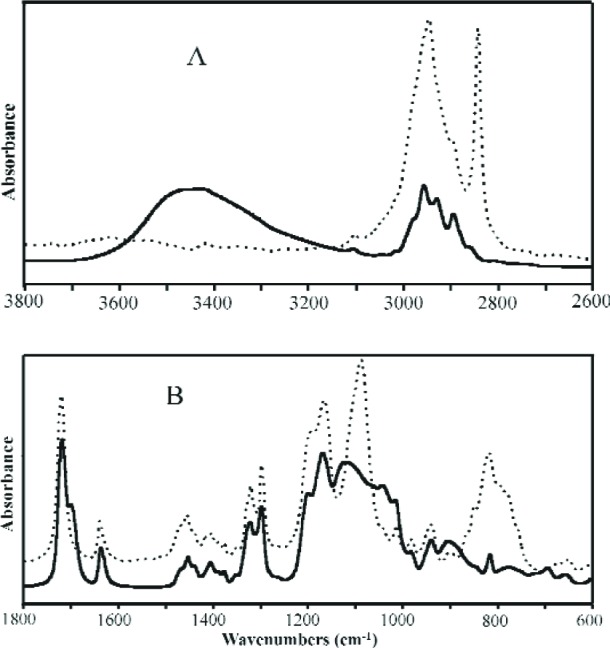
Mid-IR spectra of MPTMS (dotted line) and OMPTMS (4 weeks after start of reaction, solid line) from 3800 cm^–1^ to 2600 cm^–1^ (A) and from 1800 cm^–1^ to 600 cm^–1^ (B).

**Fig. 8:**
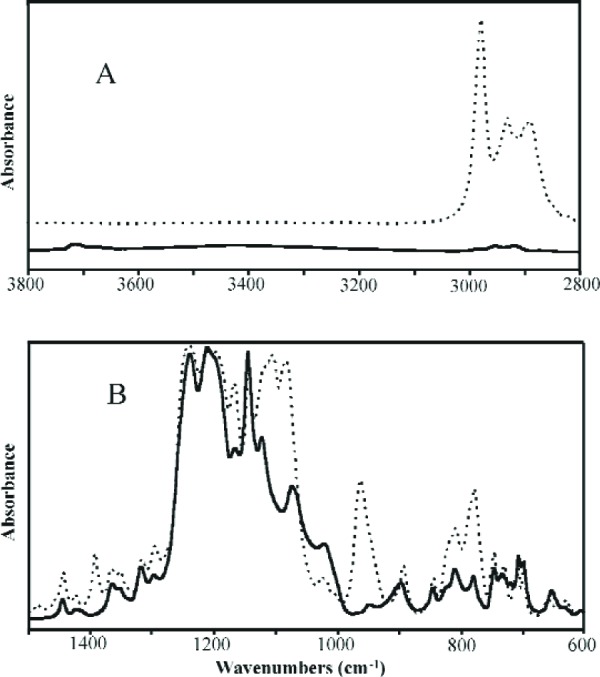
Mid-IR spectra of 13FOTES (dotted line) and O13FOTES (solid line) from 3800 cm^–1^ to 2800 cm^–1^ (A) and from 1500 cm^–1^ to 600 cm^–1^ (B).

Mid-infrared (Mid-IR) spectra of MPTMS and its condensation products are shown in Figs. 7A and 7B. The absorbances of the two Mid-IR spectra of MPTMS and OMPTMS are normalized to the C = C band at 1638 cm^–1^ (Fig. 7B); the absorbances of the spectra in Fig. 7A are expanded about two times. In Fig. 7A, the 2842 cm^–1^ band of MPTMS, CH_3_ symmetric stretch of the OCH_3_ groups, is overall absent in OMPTMS along with reduced absorbance in the asymmetric CH_3_ and CH_2_ stretch region (≈ 2950 cm^–1^). The broad band from 3800 cm^–1^ to 3000 cm^–1^ with maximum at (≈ 3450 cm^–1^ in OMPTMS is primarily ascribed to stretching of OH of Si-OH groups that are hydrogen bonded, plausibly, to O = C groups (Si-OH—O = C, Fig. 7B). Part of the 3450 cm^–1^ band may also arise from Si-OH hydrogen bonded to other Si-OH groups and from water hydrogen bonded to itself and to the Si-OH groups; however, measurements of the intensity and width of the water bending band at about 1632 cm^–1^, although difficult to measure because of the 1638 cm^–1^ C = C band, suggest less than one half the absorbance at 3450 cm^–1^ arises from water. No band near 3690 cm^–1^ is observed for “free” or non-hydrogen bonded OH of Si-OH groups. In Fig. 7B, Si-O-CH_3_ asymmetric and symmetric stretch bands of MPTMS at 1088 cm^–1^ and 817 cm^–1^, respectively, are essentially absent in the spectrum of OMPTMS, consistent with hydrolysis to form Si-OH groups and condensation of these to form Si-O-Si linkages. In the OMPTMS spectrum, the band at 904 cm^–1^ derives from an Si-(OH) stretch, the bands at 1120 cm^–1^ and 1043 cm^–1^ from Si-O-Si antisymmetric stretches, the shoulder at 1700 cm^–1^ on the C = O band at 1720 cm^–1^ from C = O that is hydrogen bonded (C = O—HO-Si), and an unassigned band at 696 cm^–1^ is tentatively assigned to an Si-O-Si symmetric stretch. Corresponding spectral changes were also observed for conversion of MDTMS to OMDTMS. We have extended the facile, low temperature syntheses of OMPTMS and OMDTMS to other types of organosilsesquioxanes. By these methods, both non-functional and multifunctional oligomers are easily derived from trialkoxyorganosilanes. The infrared spectra of a non-reactive organosilane, (tridecafluoro-1,1,2,2-tetrahydro-octyl)triethoxysilane, 13FOTES, and its silsesquioxane, O13OTES, are shown in Figs. 8A and 8B [43].

**Fig. 9:**
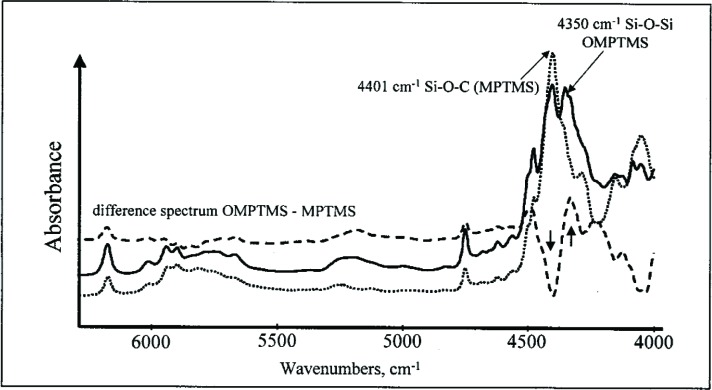
NIR spectra of MPTMS and OMPTMS. The difference spectrum demonstrates the disappearance of the Si-O-C band at 4401 cm^–1^ and the appearance of the Si-O-Si band at 4350 cm ^–1^.

**Fig. 10:**
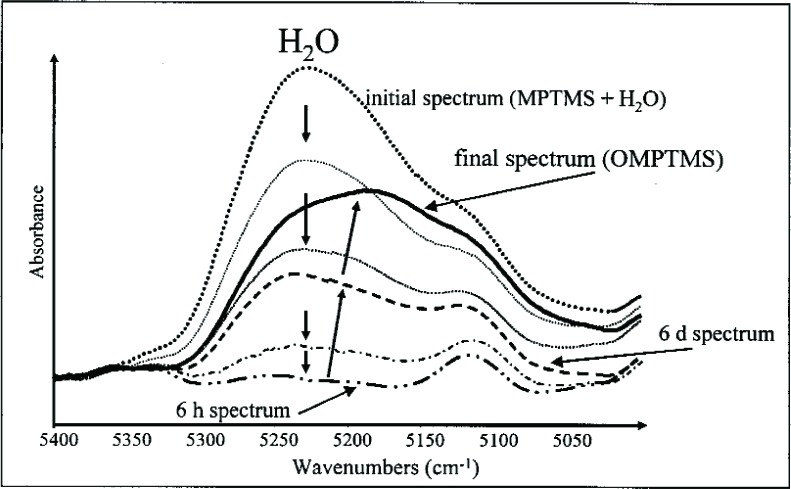
NIR spectra in the water region of MPTMS after mixing MPTMS and water. These spectra show first the consumption of water during hydrolysis followed by an increase of the water band.

The progressive chemical changes during conversion of MPTMS to the growing oligomers, loss of OCH_3_ groups on hydrolysis to form Si-OH, and condensation of Si-OH to form Si-O-Si linkages were also observed by near-infrared (NIR) spectroscopy [45]. NIR spectra (Fig. 9) from (4000 to 6500) cm^–1^ were obtained to follow the progress of the reaction and to determine qualitatively and quantitatively the fate of water contained in MPTMS, OMPTMS and the reaction mixtures. The photo-polymerization kinetics of the activated reaction products were analyzed in duplicate runs by real time (RT) NIR spectroscopy following the decrease of the =CH_2_ stretching overtone vibration (≈6167 cm^–1^). The reactions of MPTMS with water to form OMPTMS and of MPTMS with Bis-GMA or a Bis-GMA/TEGDMA mixture were studied under similar conditions. In the NIR region, the formation of OMPTMS was indicated by a decrease of a band at about 4401 cm^–1^ assigned to the alkoxysilane groups of MPTMS (Si-O-C-H_3_) and the appearance of a new band at 4351 cm^–1^ due to newly formed Si-O-Si bonds. Following the reaction of MPTMS in the presence of a small amount of water (mass fraction of 2.6 %), it was found that the water began to decrease within 2 min after mixing, indicating the immediate onset of hydrolysis of alkoxy groups to silanol groups (Fig. 10). After 6 h, the amount of water started to increase as a consequence of further silanol polycondensation reactions.

**Fig. 11:**
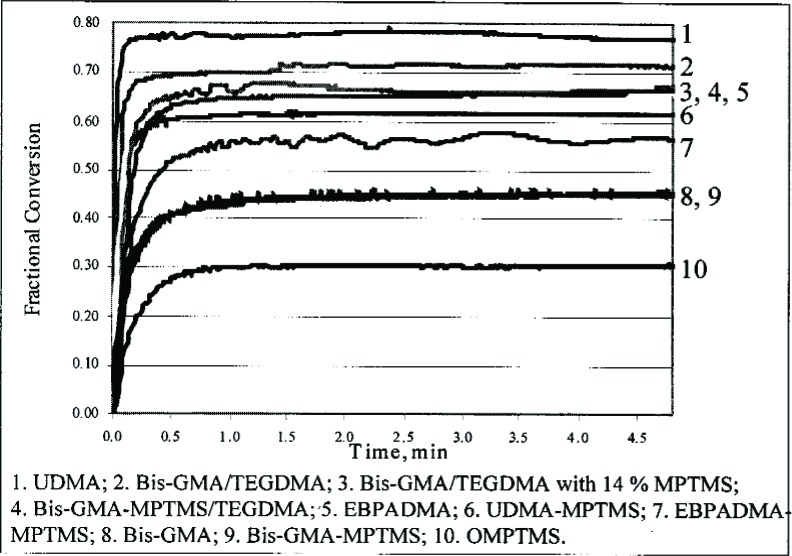
Curing kinetics of various base resin/MPTMS reaction products followed by real-time NIR spectroscopy.

Deconvolution of the water bands (not shown) indicated that water in MPTMS exists mostly as free water, while in OMPTMS only 30 % exists as free water. The amount of water in the reaction product was found to be slightly higher than in the starting materials [45]. This study also evaluated the reactions of MPTMS with non-hydroxylated dental monomers, e.g., ethoxylated bisphenol A dimethacrylate (EBPADMA) and 1,6-bis(methacryloxy-2-ethoxycarbonylamino)-2,4,4-trimethylhexane (UDMA, an aliphatic diurethane dimethacrylate derived from the reaction of 2-hydroxyethyl methacrylate and 2,4,4-trimethyl-1,6-diisocyanatohexane). The photo-polymerization kinetics of the reaction products were followed by real-time NIR spectroscopy (Fig. 11). For comparison, the reactions of MPTMS, and MPTMS with Bis-GMA or a Bis-GMA/TEGDMA mixture were studied under similar conditions [45,46].

##### 3.1.6.2 Effects of OMPTMS on Dental Polymers and Composites

A preliminary evaluation of the mechanical properties of a series of resins and composites formulated with varying amounts of OMPTMS was made starting with a resin consisting of equal masses of Bis-GMA and TEGDMA [47]. A series of photoactivated Bis-GMA/TEGDMA resins containing increasing mass fractions of OMPTMS were prepared, polymerized with visible light irradiation, and their flexural strength (FS) and elastic modulus (EM) values (Table 3) determined. Similarly, silanized quartz-filled composites (mass fraction of 75 % quartz; mean filler size = 28 µm; silanized with mass fraction of 0.5 % MPTMS) based on these resins were prepared, polymerized, and their FS and EM values determined (Table 4) after 24 h storage at 37 °C in distilled water. The mean FS and EM values or the unfilled resins and their corresponding composites are summarized in Tables 3 and 4. Results were analyzed by one-way ANOVA and Scheffe’s multiple range test.

The EM values of the resin series (Table 3) and the composite series (Table 4) were not significantly different within each series at the 99 % confidence level. The FS value for the 36.3 % OMPTMS resin (Table 3) was significantly lower than that of the other resins, suggesting that at relatively high OMPTMS contents, the fracture behavior of the unfilled polymer was dominated by the highly brittle nature of the OMPTMS segments in the crosslinked polymer. The FS value for the 36.3 % OMPTMS composite (Table 3) was significantly lower than that of the other composites. However, a positive interaction between the quartz filler and the OMPTMS-containing matrix may be occurring, because for the 36.3 % OMPTMS formulation, the ratio of (composite FS)/(resin FS) is 2.08, 34 % higher than that of the Bis-GMA/TEGDMA control that has a ratio of 1.55.

#### 3.1.7 Interaction of Organosilanes With Bis-GMA and Other Dental Monomers

**Fig. 12:**
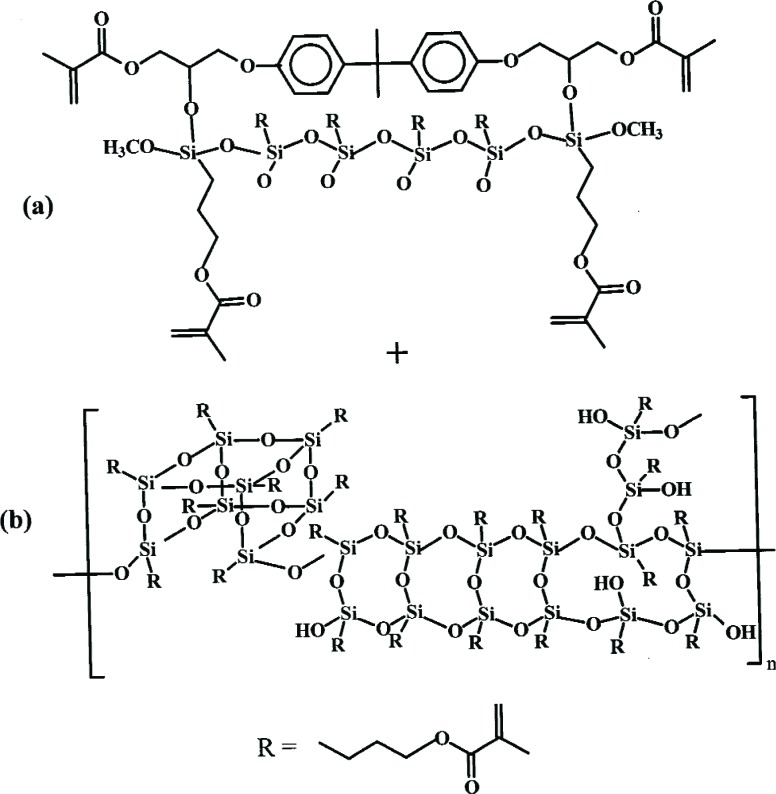
Silyl ether (a) and silsesquioxanes (b) form when the co-reactant contains hydroxyl groups and water.

**Scheme 2:**
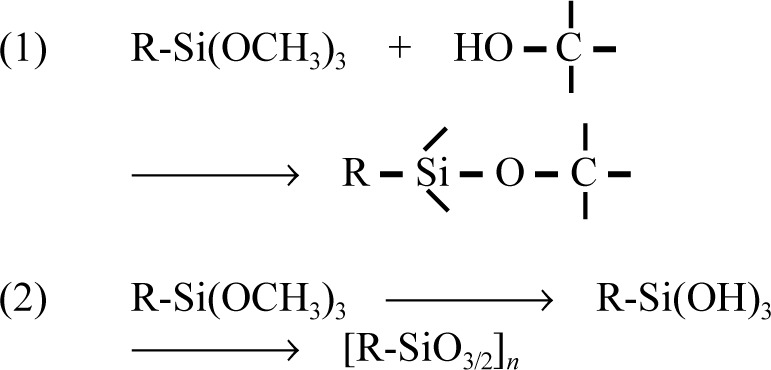
Mechanistic pathways in hydroxylated monomer/MPTMS reactions

As previously discussed, an attractive alternative to the above presilanization methods for surface activation of the filler phase of composites is *in situ* silanization of glass fillers [17,29]. This technique is simpler than presilanization and involves adding the silane agent, e.g., MPTMS, as a comonomer to the usual dental monomer systems, and then forming a composite paste by admixture with unsilanized glass filler. While it has been shown (by indirect mechanical tests) that the *in situ* silanization can affect coupling of the glass and polymer matrix phases, it is not clear that the silanization reaction only occurs between the silanol groups of the glass filler (Fig. 3) and the –Si(OCH_3_)_3_, –Si(OH)_3_, etc. groups of MPTMS. It appears plausible that there can also be exchange reactions occurring between MPTMS and the hydroxyl groups of hydroxylated monomers such as Bis-GMA in the resin [see Scheme 2, reaction (1)], resulting in the formation of silyl ether derivatives of Bis-GMA (Fig. 12) [45,46].

In addition, all polar dental monomers such as Bis-GMA, TEGDMA and UDMA have hydrophilic functional groups, e.g., hydroxyl, ethylene oxide, and urethane groups, respectively, that can serve as sites for water absorption. Because of the ubiquitous presence of water in these polar monomers, it is conceivable that MPTMS also can undergo hydrolysis-condensation reactions to form oligomeric silsesquioxane products. This indeed turned out to be the case when the interaction of MPTMS with a variety of dental monomers was studied under relatively mild conditions as described below.

The dental monomers Bis-GMA, EBPADMA, TEGDMA and UDMA were mixed neat at 22 °C with MPTMS at a mole ratio of dental monomer/MPTMS of 1.5. After a clear solution was obtained by mechanical stirring (usually within 30 min), the mixtures were heated at 60 °C in tared opened vials until no further mass loss was observed (usually a period of 31 d). Because the mass loss of TEGDMA/MPTMS was low compared to the other systems, heating was continued for an additional 30 d. The viscosities of the final products were visually compared to those of the starting mixtures. FTIR spectroscopy was used to analyze both the starting monomer/MPTMS mixtures and their final products. The interactions of *n*-propyltrimethoxysilane, allyltrimethoxysilane and vinyltrimethoxysilane with these monomers also were studied.

**Fig. 13:**
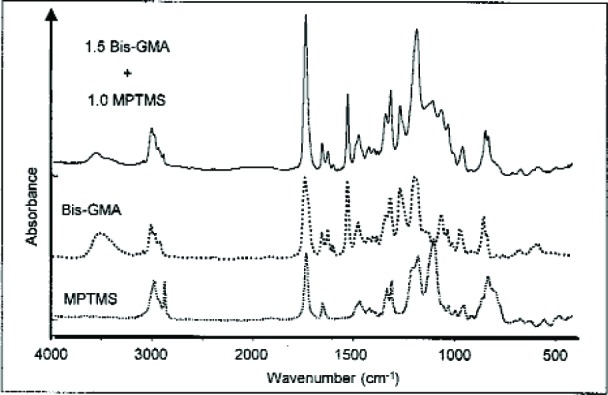
Mid-IR spectra of MPTMS, Bis-GMA and their reaction products (top spectrum).

For Bis-GMA/MPTMS the mass loss after 31 d was 9 %; a significant increase in viscosity occurred compared to that of the starting mixture. FTIR analysis (Fig. 13) of the colorless, viscous liquid product showed a significant decrease in the broad absorption band of the hydrogen-bonded hydroxyl groups of Bis-GMA in the 3650 cm^–1^ to 3150 cm^–1^ region (the band intensity changes were normalized to the Bis-GMA aromatic bands at 1608 cm^–1^, 1582 cm^–1^ and 1510 cm^–1^). In addition, the CH absorption band at 2842 cm^–1^, attributable to –Si (OCH_3_)_3_, no longer was present. New bands appeared in the 1000 cm^–1^ to 1200 cm^–1^ region that arise from Si-O-Si linkages of the silsesquioxanes products derived from the hydrolysis-condensation reactions of MPTMS, and from the expected silyl ether derivatives of Bis-GMA, although no specific C-O-Si bands were identified by Mid-IR. Thus, the overall spectral results indirectly suggest that significant amounts of the OH groups of Bis-GMA were converted to silyl ether derivatives by a transetherification exchange reaction with MPTMS and that silsesquioxane products also formed.

**Fig. 14:**
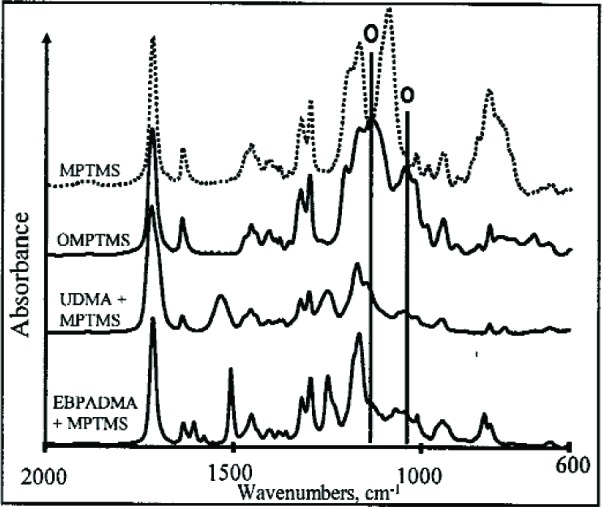
Mid-IR spectra of MPTMS the reaction products of MPTMS with water forming OMPTMS with UDMA and with EBPADMA. Vertical lines (denoted O) correspond to Si-O-Si stretching band positions.

In the case of EBPADMA/MPTMS the mass loss was about 8 % and a modest increase in viscosity accompanied this loss. Because of the absence of –OH groups and other sources of labile hydrogens in this monomer, silyl ether formation was ruled out. However, FTIR analysis again indicated virtually complete loss of the methoxy silyl group of MPTMS (2842 cm^–1^ not shown) and the appearance of absorption bands attributable to silsesquioxane formation in the region from 1000 cm^–1^ to 1200 cm^–1^ (Fig. 14). Apparently, the presence of modest amounts of water in this relatively hydrophobic monomer is still enough to convert MPTMS to silsesquioxane products via the apparently uncatalyzed hydrolysis-condensation reactions of the methyl silyl ether groups.

A similar reaction occurred with TEGDMA/MPTMS. After 31 d the pale yellow liquid had lost only about 1.3 % of its original mass and had only a slightly higher viscosity than the starting mixture. Continued heating (30 d) resulted in greater mass loss (3.5 %), but during the last days of this extra heating period it polymerized to a hard, pale yellow glassy solid. FTIR analysis of the polymer indicated that significant silsesquioxane formation had occurred in the TEGDMA/MPTMS blend.

After 31 d the mass loss of the UDMA/MPTMS product had leveled off at 10 % and a significant increase in viscosity was noted for this blend, greater than that observed with EBPADMA and TEGDMA, but less than that of the Bis-GMA/MPTMS blend. Although the highly polar structure of UDMA with two potentially labile hydrogens in its urethane group would seem to make this monomer a possible candidate for an exchange reaction with MPTMS, FTIR analysis of the reaction product mixture again showed only loss of the methoxysilyl group (2842 cm^–1^) and the presence of absorption bands attributable to silsesquioxane structure and the unchanged UDMA. Results similar to those observed with MPTMS were found to occur with the other trimethoxysilanes and Bis-GMA, EBPADMA or UDMA, respectively.

Two mechanistic pathways exist for the reaction of Bis-GMA with MPTMS: (1) silyl ether formation by an exchange or transetherification reaction involving the –Si-OCH_3_ groups of MPTMS with the hydroxyl groups of Bis-GMA and, (2) due to the presence of H_2_O in this hydroxylated dental monomer (or from the ambient atmosphere), MPTMS also can undergo a series of hydrolysis-condensation reactions via its –Si(OCH_3_)_3_ groups, leading to silsesquioxane formation. Scheme 2 depicts the two possible pathways for these reactions.

The first and second mechanistic pathway applies to the interaction of these silanes with Bis-GMA only. The second mechanistic pathway prevails with TEGDMA, EBPADMA and even the more highly polar UDMA, (that has potentially polarizable urethane groups), where only silsesquioxane formation was observed. These results are similar to those from a previous study in which it was demonstrated that polymeric silsesquioxanes such as those derived from MPTMS could be obtained by hydrolysis-condensation reactions in aqueous acetone without a catalyst [44]. The interaction of these dental monomers with other trialkoxysilanes, such as *n*-propyltrimethoxysilane, vinyltrimethoxysilane and allyltrimethoxysilane, occurs by the same mechanistic pathways. In the reaction of Bis-GMA with the silane, the predominant products were in every case silyl ether derivatives, whereas EBPADMA/TEGDMA and UDMA served only as polymerizable solvents for the *in situ* formation of oligomeric silsesquioxanes [45,46]. The water content of the monomer also may be a factor in controlling the amount of silsesquioxanes formed from the silane. For example, in contrast to Bis-GMA, a recent study with the more hydrophilic glyceryl methacrylate containing (in a mole ratio of 1.5) MPTMS yielded more oligomeric silsesquioxane products than silyl ether derivatives, presumably because of the greater water content of glyceryl monomer. These reactions of silanes directly with hydroxyl groups of monomers or with water present in non-hydroxylated monomer can provide facile routes to novel types of dental resins that can combine acrylic and silicon chemistries.

Thus, the exchange reaction of MPTMS, and similar organotrialkoxysilanes, to form silyl ether derivatives of dental monomers requires the presence of hydroxyl groups or similar protic functional groups with labile hydrogen. However, polar, but non-hydroxylated monomers can serve as polymerizable solvents for the *in situ* generation of oligomeric silsesquioxanes from MPTMS (and other organotrialkoxysilanes) by sequential hydrolysis-condensation reactions induced by the presence of ambient water only.

## 4. Conclusions

Organosilanes, especially functional organosilanes, have a remarkable, versatile chemistry that extends well beyond their use as surface treatment agents for mineral substrates. Because of the unique dual functionality of silane coupling agents, they can form chemical bridges that unite disparate organic and inorganic materials as exemplified by their use in creating an adhesive interphase in silica-reinforced polymeric dental composites. Studies indicate that the strength and durability of this interphase is dependent on the chemical structure of the silane agent(s) and the silanization process, but more structure-property studies are needed to better elucidate the nature of this interface.

Organotrialkoxysilanes in the presence of water (even just ambient moisture) are capable of a complex series of hydrolysis-condensation reactions that can lead to three-dimensional silsesquioxane structures. Functional organotrialkoxysilanes can easily be converted to reactive oligomeric or polymeric silsesquioxanes that have potential both as resin-matrix components and as molecular-sized, organically modified silica fillers.

In addition to their self-condensation reactions, organosilanes are capable of exchange reactions with hydroxylated or carboxylated dental monomers and oligomers to form high molecular mass silyl derivatives. Because of the ubiquitous presence of ambient moisture in these resins *in situ* formation of oligomeric/polymeric silsesquioxanes also occurs. Blends of silyl derivatives and silsesquioxanes in dental resins are expected to have beneficial effects with regard to ameliorating the effects of polymerization shrinkage and stress development in composites.

## Figures and Tables

**Table 1:** Flexural strength, mean and standard deviation, in MPa

		Quartz, MPTMS	Quartz, MDTMS	Glass, MPTMS	Glass, MDTMS
37 °C,	24 h	123 (4)^a^	126 (11)	88 (12)	98 (15)
90 °C,	408 h	95 (6)	109 (8)	57 (4)	79 (11)

a Numbers in parentheses indicate one standard deviation as an estimate of the standard uncertainty.

**Table 2:** Diametral tensile and flexural strength (MPa) of bis-GMA/TEGDMA (1:1 by mass) composites with varying amounts of a silica filler

Silane coupling agent	Silica-filler volume fraction (%)	Diametral tensile strength	Flexural strength	
		24 h	24 h	2 wk
MPTMS	67	57 (3)^a^	100 (10)	80 (4)
MDTMS	67	62 (3)	103 (16)	95 (8)
MDTMS	71	61 (3)	118 (13)	105 (9)
MDTMS	74	64 (3)	137 (7)	– – –

a Numbers in parentheses indicate one standard deviation as an estimate of the standard uncertainty. Number of specimens > 5; Specimens stored in distilled water at 37 °C for indicated times. Photoinitiator system: 0.2 % (mass fraction) camphorquinone, 0.8 % (mass fraction) ethyl 4-dimethylaminobenzoate.

**Table 3:** Resin flexural strength, FS, (MPa) and elastic modulus, EM, (GPa)

Mass fraction % OMPTMS in Bis-GMA/TEGDMA	FS	EM
0 (control)	88.0 (8.6)^a^	2.3 (0.1)
9.1	83.0 (11.8)	2.4 (0.1)
18.2	74.1 (7.5)	2.3 (0.1)
36.3	45.1 (7.8)^b^	2.3 (0.1)

a Numbers in parentheses indicate one standard deviation as a measure of standard uncertainty; *n* = 10.

b Values in each series are significantly different at the 99 % confidence level.

**Table 4:** Composite flexural strength, FS, (MPa) and modulus, EM, (GPa)

Mass fraction % OMPTMS in bis-GMA/TEGDMA	FS	EM
0 (control)	136.2 (11.8)^a^	13.3 (1.0)
9.1	136.8 (9.9)	14.3 (0.6)
18.2	129.4 (6.3)	13.9 (0.8)
36.3	93.8 (12.9)^b^	13.6 (0.6)

a Numbers in parentheses indicate one standard deviation as a measure of standard uncertainty; *n* = 10.

b Values in each series are significantly different at the 99 % confidence level.
